# Conventional mechanical ventilation

**DOI:** 10.4103/1658-354X.65128

**Published:** 2010

**Authors:** Joseph D. Tobias

**Affiliations:** *Department of Anesthesiology & Pain Medicine, Nationwide Children's Hospital, Columbus, Ohio*

**Keywords:** *Mechanical ventilation*, *respiratory failure*, *ventilatory support*

## Abstract

The provision of mechanical ventilation for the support of infants and children with respiratory failure or insufficiency is one of the most common techniques that are performed in the Pediatric Intensive Care Unit (PICU). Despite its widespread application in the PICUs of the 21st century, before the 1930s, respiratory failure was uniformly fatal due to the lack of equipment and techniques for airway management and ventilatory support. The operating rooms of the 1950s and 1960s provided the arena for the development of the manual skills and the refinement of the equipment needed for airway management, which subsequently led to the more widespread use of endotracheal intubation thereby ushering in the era of positive pressure ventilation. Although there seems to be an ever increasing complexity in the techniques of mechanical ventilation, its successful use in the PICU should be guided by the basic principles of gas exchange and the physiology of respiratory function. With an understanding of these key concepts and the use of basic concepts of mechanical ventilation, this technique can be successfully applied in both the PICU and the operating room. This article reviews the basic physiology of gas exchange, principles of pulmonary physiology, and the concepts of mechanical ventilation to provide an overview of the knowledge required for the provision of conventional mechanical ventilation in various clinical arenas.

## INTRODUCTION

The support of infants and children with respiratory failure or insufficiency through mechanical ventilation is one of the most common techniques or procedures performed in the Intensive Care Unit (ICU). Despite its widespread application in the ICUs of the 21^st^ century, before the 1930s, respiratory failure was uniformly fatal due to the lack of equipment and techniques for airway management and ventilatory support. The first widespread use of mechanical support for respiratory failure began with negative pressure ventilation during the poiliomyelitis epidemic of the 1930s (see below for a discussion of negative pressure ventilation). The operating rooms of the 1950s and 1960s provided the arena for the development of the manual skills and the refinement of the equipment needed for airway management, which subsequently led to the more widespread use of endotracheal intubation thereby ushering in the era of positive pressure ventilation. Although mechanical ventilation remains a commonly used technique in both the operating room and the ICU, all of us at some point are or were filled with trepidation and uncertainty when faced with initiating mechanical ventilation. Although, the devices used to provide ventilatory support and the various clinical decisions to be made will initially seem overwhelming, a logical approach to the provision of mechanical and its initial set-up will help in the decision-making process. The approach to the provision of mechanical ventilation in the critically ill patient is supported by an understanding of the basics of pulmonary physiology and gas exchange.

## RESPIRATORY PHYSIOLOGY AND MECHANICAL VENTILATION

### Indications for mechanical ventilation

Although there are a diverse group of disease processes involving the central nervous and peripheral nervous systems, cardiovascular, and respiratory systems, which may lead to respiratory failure, there are a limited number of primary indications for the institution of endotracheal intubation and mechanical ventilation in the ICU setting [Table 1]. As the severity of the primary lung injury is extremely variable in these categories, so will be the type of mechanical ventilatory support that is required. In fact, some patients who have normal respiratory function may require airway control and mechanical ventilation due to neuromuscular weakness or “…altered mental status."

**Table 1:** Indications for airway control and mechanical ventilation

Pulmonary parenchymal disease (adult respiratory distress syndrome, pneumonia)
Airway problems (croup, epiglottitis, upper airway obstruction)
Lower airway obstruction (asthma)
Lowered mental status (intoxication, traumatic brain injury)
Neuromuscular weakness (Guillain–Barre, botulism, inadequate diaphragmatic blood flow shock)

### Etiology of hypoxemia

Respiratory insufficiency may result primarily in hypoxemia, hypercarbia, or a combination of the two. When confronted with the hypoxemic patient (it is imperative when discussing respiratory physiology to differentiate between hypoxemia, a low oxygen content or saturation in the blood, and hypoxia which is inadequate oxygen at the tissue level), the treatment will be tailored according to the etiology of the hypoxemia. In clinical practice, there are five basic causes of hypoxemia [Table 2]. In clinical practice, a low fraction of inspired concentration of oxygen (FiO_2_) is rare, but may be encountered while providing care at higher altitudes, if there is some mechanical issue with the hospital's oxygen supply, or if there is a mismatch of the appropriate flows of gases used either in the operating (oxygen and nitrous oxide) or in the ICU setting (oxygen and helium).[1] Although a low FiO_2_ as the primary cause of hypoxemia should never occur in the context of modern medical care, it is imperative that the FiO_2_ be continuously monitored when medical gases are mixed. The other four processes leading to hypoxemia may be seen in the ICU setting. True shunt (as opposed to the shunt seen in ventilation–perfusion inequalities) refers to patients with blood that travels from the right (venous) side of the circulation to the left (arterial) side without traversing the lungs. Although there is a normal 2–3% obligatory physiologic shunt which includes both the bronchial veins that empty into the left atrium and the Thebesian veins (venous drainage of the coronary system which drain into the left ventricular cavity), this has no significant impact on the arterial oxygen saturation. What occurs in clinical practice is cyanotic congenital heart disease (CHD) such as Tetralogy of Fallot, tricuspid atresia, pulmonary atresia resulting in right-to-left intracardiac shunting of blood. This cause of hypoxemia does not generally show a significant response or increase in the partial pressure of oxygen in the blood (PaO_2_) when the FiO_2_ is increased. The failure to respond to an increasing FiO_2_ can be used as a diagnostic test when evaluating the cyanotic newborn as an increase in the PaO_2_ to some degree will occur even in patients with hypoxemia from severe pulmonary parenchymal disease. Although surgical intervention may be necessary in patients with true shunt from CHD to provide adequate pulmonary blood flow, these patients generally do not present with clinical signs and symptoms of respiratory failure and tend to tolerate oxygen saturations in the 70–80% range without manifesting dyspnea or other signs of respiratory failure. Therefore, clinically it may be possible to distinguish the newborn with cyanosis from CHD versus those with pulmonary parenchymal disease, based on the fact that signs of respiratory distress are generally less obvious in the setting of CHD provided there is no accompanying congestive heart failure.

**Table 2:** Etiology of hypoxemia

Low inspired oxygen concentration
True shunt
Ventilation–perfusion mismatch
Diffusion abnormalities
Hypoventilation

Another cause of hypoxemia, also sometimes inappropriately referred to as shunt, is ventilation–perfusion mismatch. This refers to processes such as pneumonia, adult respiratory distress syndrome, or atelectasis that disrupts the normal gas exchange occurring at the alveolar–capillary level with the exposure of pulmonary capillary blood to poorly ventilated alveoli. Ventilation–perfusion mismatch refers to perfusion of poorly ventilated alveoli. The opposite of the process, ventilation without perfusion or deadspace, results in hypercarbia generally without hypoxemia. One of the goals of mechanical ventilation is to improve the matching of ventilation and perfusion to improve oxygenation through the use of recruitment maneuvers if the cause is atelectasis, or the application of positive end expiratory pressure (PEEP) if the cause is adult respiratory distress syndrome (ARDS) or other alveolar diseases (see below). Hypoxemia may also result from diffusion abnormalities from a process, which affects the pulmonary endothelial–basement membrane–alveolar epithelial complex, thereby interfering with the diffusion of oxygen (O_2_) into and carbon dioxide (CO_2_) out of the alveoli. This latter process is generally rare in the ICU setting, occurring most commonly in conditions that thicken these layers through which O_2_ and CO_2_ must diffuse. Causes include collagen vascular disorders, sarcoidosis, and other diseases that affect the endothelial–basement membrane–epithelial layer of the alveolus. The final of the five causes of hypoxemia is hypoventilation. Associated with the hypoxemia of hypoventilation is hypercarbia, which is relatively uncommon in other causes of hypoxemia except with severe forms of ventilation–perfusion inequalities and diffusion abnormalities.

### Respiratory failure and mixed venous oxygen saturation

Another physiologic issue of importance when caring for the hypoxemic patient with ventilation–perfusion mismatch or true shunt is the impact that the saturation of the mixed venous blood has on the eventual arterial saturation. As the blood passes through the capillary systems of non-ventilated alveoli, no additional oxygen is picked up and therefore the oxygen saturation of the blood in the pulmonary venous capillary system is equivalent to the blood when it started its journey through the pulmonary vascular blood (i.e., mixed venous saturation). Therefore, therapy that improves mixed venous oxygen saturation may also improve arterial oxygen saturation in patients with significant ventilation–perfusion mismatch or true shunt. Mixed venous oxygen saturation is determined by the difference between the oxygen that is delivered to the tissues and the amount of oxygen used by the tissues. Methods to improve oxygen delivery include increasing the hemoglobin concentration or augmenting cardiac output while preventing hyperthermia, shivering, or agitation, which increase tissue oxygen consumption. These strategies may be of significant clinical application in patients with severe respiratory failure and refractory hypoxemia.

### The alveolar–arterial oxygen gradient

In the setting of hypoxemia, an evaluation of the severity of the lung disease can be determined based on the difference between the PaO_2_ and the partial pressure of oxygen in the alveoli. This is commonly referred to as the A–a or the alveolar–arterial oxygen gradient. The normal value of 10–15 mmHg frequently exceeds 200 mmHg in the critically ill patient with respiratory failure. The alveolar partial pressure of oxygen is determined using Dalton's law which states that the gases in the closed space of the alveolus must equal barometric pressure (generally assumed to be 760 mmHg). There are generally only four gases in the alveolus including O_2_, nitrogen (unless the FiO_2_ is 1.0), water vapor (partial pressure = 47 mmHg at 37°C), and CO_2_. Although in the strictest sense, the alveolar pressure of CO_2_ is calculated as the PaCO_2_/RQ, where RQ is the respiratory quotient (generally 0.8 depending on the intake of fat and carbohydrate), for clinical purposes, the alveolar pressure of CO_2_ can be assumed to be equivalent to the partial pressure of CO_2_ in the blood (PaCO_2_), thereby resulting in the equation: alveolar oxygen concentration = FiO_2_ (760 – 47) – PaCO_2_. As the alveolar partial pressure of O_2_ is approximately linear with the FiO_2_, a quick estimate of the alveolar oxygen concentration can be had by multiplying the oxygen concentration (percentage) by 60–70.

### Physiology of oxygenation and ventilation

The primary goals of mechanical ventilation are the maintenance of adequate oxygenation and clearance of CO_2_ from the body in the amount needed to maintain cellular homeostasis. Oxygenation is regulated by the FiO_2_ and the mean airway pressure. Mean airway pressure is determined by the peak inflating pressure (PIP), PEEP, and the inspiratory time.[2] Increasing the mean airway pressure by manipulation of any of the three previously mentioned variables recruits alveoli, improves ventilation–perfusion matching, and decreases intrapulmonary shunting. In addition to reducing ventilation–perfusion inequalities and increasing functional residual capacity (FRC), increasing mean airway pressure may also result in a significant improvement in respiratory compliance, thereby allowing for more effective spontaneous ventilation and decreasing the PIP required to provide adequate tidal ventilation, and hence limits the potential for barotrauma during mechanical ventilation.(3)

One of the goals of mechanical ventilation, regardless of the mode and setting, is the restoration of FRC. Critical in the maintenance of a normal ventilation–perfusion ratio is the relationship between FRC and closing capacity (CC). CC is the lung volume at which small airway closure occurs during expiration. Conditions that decrease FRC below CC or increase CC above FRC result in a maldistribution of ventilation/perfusion and adversely affect the mechanics of breathing and ventilation [Table 3]. In the school-aged children and adults, FRC is normally greater than CC. However, in young infants and toddlers, CC may exceed FRC even in the healthy state.[4] Conditions associated with a decreased FRC (i.e., pulmonary edema, pneumonitis, infant and acute respiratory distress syndromes) are treated with PEEP to increase FRC back to normal levels (see below for a full discussion regarding PEEP). Situations associated with increased CC (i.e., bronchiolitis, reactive airway disease) are treated with bronchodilators and measures to control secretions to reduce CC and maintain airway patency.

**Table 3:** Factors affecting closing capacity and functional residual capacity

Increased closing capacity
Infancy
Bronchiolitis
Asthma
Bronchopulmonary dysplasia
Smoke inhalation with thermal injury to airway
Cystic fibrosis
Reduced functional residual capacity
Supine position
Abdominal distention
Obesity
Thoracic or abdominal surgery or trauma
Atelectasis
Pulmonary edema
Acute lung injury/ ARDS
Near drowning
Aspiration pneumonia
Infectious pneumonia
Radiation pneumonitis

Effective mechanical ventilation also provides minute ventilation, calculated as the respiratory rate (RR) times the tidal volume (*V*_T_) that is adequate for CO_2_ removal. The PaCO_2_ is directly related to the body's production of CO_2_ during the catabolism of fats and carbohydrates and inversely related to alveolar ventilation. In most clinical circumstances, the control of PaCO_2_ will rely on alterations in the minute ventilation; however, some control of the body's endogenous CO_2_ production is possible through the increase in the use of fats versus carbohydrates for nutrition or by the control of body temperature. As the respiratory quotient (CO_2_ production/O_2_ consumption) for carbohydrates is 1 versus 0.7 for fats, an increased reliance on fats to provide caloric requirements can be used to limit endogenous CO_2_ production and thereby minimize ventilatory requirements. Furthermore, prevention of hypothermia and even induction of mild hypothermia (35°C) can also be used clinically to control hypercarbia and limit mechanical ventilatory requirements. It must also be stressed that in patients with severe lung disease, ventilation to normocarbia is not necessary and may in fact be harmful. Current practice includes the use of permissive hypercarbia or allowing the PaCO_2_ to increase provided the pH is kept above 7.25. This strategy has been shown to improve outcome in patients with ARDS.[5] Although generally advocated in the treatment of ARDS and other types of severe lung injury, the end-organ effects of hypercarbia must be considered. These may include detrimental effects on cardiovascular function and increases in cerebral blood flow and intracranial pressure. As such, the degree of hypercarbia tolerated will depend on the presence of comorbid conditions.

Although minute ventilation is defined as RR times VT, the entire VT is not involved in the effective gas exchanged. The part of the VT that does not participate in gas exchange is referred to as physiologic deadspace. Total or physiologic deadspace is composed of anatomic deadspace (the area of the conducting areas or the trachea and bronchi that do not participate in gas exchange) and alveolar deadspace (those alveoli which are ventilated but not perfused). In the healthy state, the alveolar deadspace is minimal so that anatomic and physiologic deadspaces are approximately the same. Although anatomic deadspace, representing approximately 30% of a normal tidal breath or 150 mL in an average-sized adult, does not generally change regardless of the disease process, alveolar deadspace may change significantly in patients with pulmonary parenchymal disease, pulmonary vascular disease, or with changes in cardiac output resulting in alterations in pulmonary perfusion. The latter principle is clearly demonstrated by the abrupt decline in end-tidal CO_2_ (ETCO_2_) that occurs with cardiac arrest, a decrease in cardiac output, or pulmonary embolism. The measurement of physiologic deadspace can be performed using Bohr's method. This is based on the principle that all exhaled CO_2_ comes from alveoli that are perfused since deadspace does not receive pulmonary perfusion and is therefore devoid of CO_2_. The Bohr equation states: VD/VT = (PaCO_2_ – PECO_2_)/PaCO_2_ where VD is the deadspace ventilation, VT the tidal volume, and PECO_2_ the partial pressure of CO_2_ in mixed expired gas.

Since the anatomic deadspace (VD) is relatively constant in patients with healthy lungs, increasing the VT decreases the ratio of VD to VT. In effect, the VT increases alveolar ventilation. In patients with intrinsic lung disease undergoing mechanical ventilation, there is ventilation of poorly perfused regions of the lungs (alveolar VD). In this setting, increases in VT may not decrease VD/VT since higher alveolar pressures due to larger VT may result in a further decrease in pulmonary perfusion and an increase in alveolar VD. An estimation of the effect of VT changes on VD/VT in such clinical scenarios can be provided by estimating VD/VT using capnography with ETCO_2_ measurements and the following equation:

VD/VT = (PaCO_2_ – PETCO_2_)/PaCO_2_ In summary, it can be determined that a change in the metabolic rate with an alteration in CO_2_ production, a change in minute ventilation (RR or VT), or a change in VD may affect PaCO_2_.

## NEGATIVE PRESSURE VENTILATION

**Figure 1:**
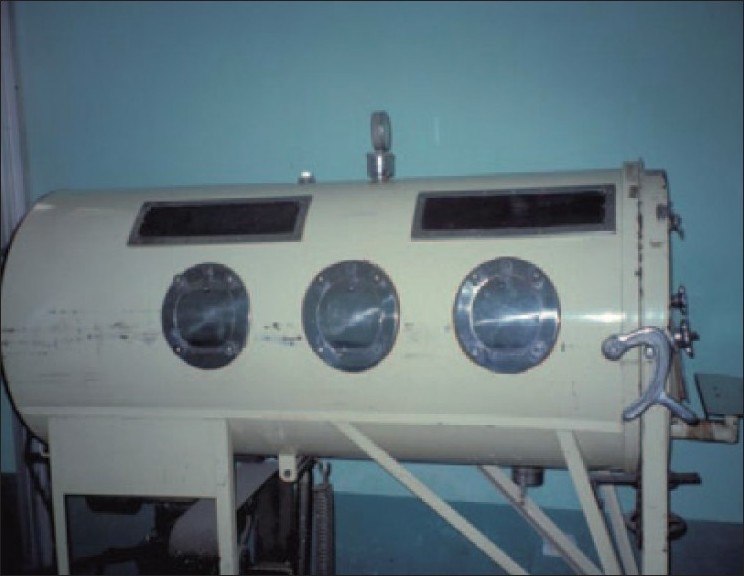
Photograph of a negative pressure ventilator otherwise known as the “iron lung.” These devices were used during the poliomyelitis epidemics of the 1930s and 1940s for the treatment of acute and chronic respiratory failures. Subatmospheric pressure was generated by a piston on the bottom of the device, which increased the total volume of the container without a change in the volume of the gas in the space. The magnitude of the negative pressure generated could to some extent be changed by altering the movement of the piston

With the poliomyelitis epidemic of the 1930s, negative pressure ventilation was introduced to support patients with neuromuscular weakness leading to acute and chronic respiratory failure. The negative pressure ventilators ("iron lungs") were large tanks into which the patient's entire body was placed [Figure 1]. The patient's neck was surrounded by a rubber matt with a small opening in the center through which the patient's head protruded. The driving force behind negative pressure ventilation was a piston located at the bottom of the tank. With a downward movement of the piston and an increase in the content of the iron container without an increase in the gas volume, negative pressure was created around the outside of the patient's thorax resulting in the expansion of the patient's chest wall and air entry through the mouth and nose into the patient's lung. The magnitude of the pressure change was measured from a pressure gauge situated on the top of the device. To some extent, the degree of negative pressure generated could be increased by increasing the downward movement of the piston. Although somewhat effective in patients with respiratory failure related to muscle weakness, there were significant limitations in the amount of negative pressure that could be generated, and as such, these devices were not effective in patients with significant alterations in respiratory compliance or resistance. Additionally, the devices were bulky, providing restricted access to the patient (obtained through side or port holes in the device), they offered no protection from aspiration in patients with bulbar involvement, and could not used in patients with respiratory failure related to airway disease. A major concern regarding restricted patient access in the classic iron lung is that without a specialized laryngoscopy blade ("polio blade"), endotracheal intubation while the patient is in the iron lung may be impossible.

Although not in common clinical use today, these devices hold a place in our medical history as the first artificial ventilators used on a widespread scale for patients with respiratory failure. However, the idea of negative pressure ventilation is not dead; cuirass or vests that fit over the patient's thorax and are sealed at the waist and neck are occasionally used for the treatment of acute or chronic respiratory failure in infants and children.[6] These devices initially included the Tunnicliffe jacket and the Pulmon-wrap, which used a framework of plastic or metal that fits over the patient's thorax and was covered with an airtight material with seals around the neck, arms, and thighs. The air within the jacket was intermittently evacuated thereby creating a negative pressure (compared to the atmosphere) and providing or augmenting air exchange. These devices could be used for home care and found their greatest use in patients with chronic respiratory insufficiency due to neuromuscular weakness. The next generation of negative pressure ventilation at home was ushered in with the introduction of the Hayek oscillator.[7] The device comes in a wide range of sizes that can be used from preterm infants to adults. The cuirass is attached to a piston pump that can be used at conventional frequencies (up to 120 breaths/minute or 2 Hz) to provide conventional types of ventilation. When set at higher rates (up to 15 Hz), the Hayek oscillator provides gas exchange similar to conventional high-frequency oscillatory ventilation.[7]

The advantages of negative pressure ventilation devices are that they do not require endotracheal intubation, can be applied intermittently, can be used at home without the need for tracheostomy, and the interpleural pressure decreases from the beginning to the end of inspiration (as opposed to the increase in intrapleural pressure that occurs with positive pressure ventilation). An increase in interpleural pressure can decrease venous return (preload) and cardiac output. With a decrease in interpleural pressure during negative pressure ventilation, venous return and cardiac output increase thereby matching the phasic changes that occur in these variables during normal spontaneous ventilation. While most patients tolerate the negative cardiovascular effects of positive pressure ventilation without a clinically significant effect, positive pressure ventilation may have adverse clinical effects in specific populations. One such group is children following cavopulmonary anastomoses (Fontan procedures). In these patients, the anatomical presentation of a single ventricle mandates the re-routing of blood directly from the venous circulation (superior and inferior venacavae) to the pulmonary circulation to avoid chronic long-term volume overload, which would result if the single ventricle were to pump to both the circulations. In the performance of these procedures, the (SVC) Superior Vena Cava; and subsequently, the (IVC) Inferior Vena Cava are directly anastomosed to the pulmonary artery. In the postoperative setting (both acute and long-term), positive pressure ventilation by decreasing venous return can significantly decrease pulmonary blood flow which is dependent on the passive flow of blood from the venous to the pulmonary circulation. Although current clinical practice is to attempt early tracheal extubation, negative pressure ventilation using a cuirass around the patients has been evaluated in the immediate postoperative period following cavopulmonary (Fontan) anastamosis. Shekerdemian *et al*. demonstrated that the switch from positive pressure to negative pressure ventilation resulted in an immediate mean increase in pulmonary blood flow of 42% and a total increase of 54%.[8] The improvement in pulmonary blood flow was achieved by an increase in stroke volume as no change in heart rate was noted. The increase in pulmonary blood flow disappeared with the reinstitution of positive pressure ventilation.

## POSITIVE PRESSURE VENTILATION

The era of positive pressure mechanical ventilation began with controlled mandatory ventilation (CMV) which provided intermittent positive pressure breaths to the patient without the ability to sense the patient's own respiratory efforts, with no gas flow in between the ventilator breaths, and has no means to allow the patient to breathe spontaneously thereby resulting in significant patient–ventilator asynchrony unless deep levels of sedation or neuromuscular blockade were used. An additional issue with CMV was the recognition that controlled ventilation in the absence of spontaneous breathing rapidly leads to atrophy of respiratory muscles.(9) CMV was followed by intermittent mandatory ventilation (IMV) which provided a set number of breaths per minute provided at a specific interval (if the rate were set at 12, a breath would be delivered every 5 seconds), but also allowed for spontaneous ventilation through the use of a continuous gas flow, a reservoir bag, or a demand valve. Despite the ability to allow spontaneous ventilation, the IMV mode did not synchronize the ventilator breath with the patient's effort, and therefore, it was possible that a ventilator breath could be delivered during the exhalation phase of the patient's spontaneous breath. Additionally, there was no assistance with the spontaneous breaths to overcome the work of breathing (WOB) imposed by the endotracheal tube (ETT) and the ventilator.

Given these issues, strict IMV is not used in clinical practice and is having been replaced by modes such as assist control (AC) and synchronized intermittent mandatory ventilation (SIMV) which attempt to deliver a ventilator breath that is coordinated with the patient's own inspiratory effort. Alternatively, a breath is delivered if the patient does not initiate a breath within a preset period of time. To accomplish this, the ventilator must have a way of sensing the patient's own inspiratory effort. In most clinical circumstances, this is accomplished by sensing a pressure change in airway pressure (usually –1 to –3 cm H_2_ O) within the breathing circuit of the ventilator system. Setting this value at too large a value (more than –3 cm H_2_O) may lead to failure to sense the patient's own spontaneous breath thereby leading to breathing without assistance resulting in an increased WOB (see below). Setting this value too low or making it too sensitive can lead to autocycling of the ventilator due to pressure changes caused by cardiac oscillations or a leak around an uncuffed ETT. In the latter scenario, as there is a leak around the tube, there is a loss of the PEEP and therefore a drop in the airway pressure, which may be interpreted by the ventilator as the patient's inspiratory effort. Given these issues, some adjustments of the sensitivity, based on the patient's characteristics, may be necessary.

When the decision has been made to initiate mechanical ventilation, the clinician will initially have to take the following decisions: (a) the mode of ventilation such as AC, SIMV, or pressure-regulated volume-controlled (PRVC); (b) the limit variable (pressure or volume) which will control the tidal breath and its magnitude (set as either a pressure above PEEP or a specific VT); (c) the inspiratory time; (d) the ventilator rate (breaths/minute); (e) the FiO_2_; and (f) the PEEP. The limit variable (pressure or volume) will give the name to the type of ventilation chosen: pressure-limited or volume-limited.

### Mode of ventilation

Breathing during mechanical ventilation can be controlled, assisted, supported, or spontaneous. Controlled breaths are provided regardless of the patient's respiratory efforts (eg., CMV or IMV), whereas assisted and supported breaths are synchronized with the patient's own respiratory effort. Spontaneous breaths occur without ventilator assistance and since they impose the WOB, they are generally not allowed in the modern era of mechanical ventilation. Instead, when the SIMV mode is used, spontaneous breaths are frequently supported with either pressure or volume support (see below).

When considering assisted ventilation, the basic modes of ventilation include AC, SIMV, and PRCV. With AC, the ventilator delivers full support (either pressure or volume) every time the patient initiates a breath. If the patient fails to breathe, the ventilator will deliver a fixed number of breaths per minute according to the preset rate. The theoretical advantage of AC ventilation is that every patient initiated breath is supported and the patient determines the RR. With an intact central control of respiration, the PaCO_2_ should be maintained within the normal range; however, there are many factors other than central control of ventilation which may control the RR so that tachypnea resulting in hypocarbia may occur in sepsis, central nervous system disorders, pain, and agitation. Additionally, although AC ventilation ensures support with every breath thereby limiting the WOB, when the decision is made to wean the ventilatory support, the volume or pressure of the tidal breath must be weaned and not necessarily the rate. With the AC mode of ventilation, if the rate is set at 20 breaths/minute and the patient is breathing at 30 breaths/minute, decreasing the rate to 15 breaths/minute will have an impact neither on the amount of support provided to the patient nor on the minute ventilation.

With SIMV, a set number of breaths per minute are synchronized with the patient's respiratory effort and the full support (pressure or volume) is delivered. If the patient breathes above the preset number of breaths each minute, there will be additional minute ventilation from this spontaneous ventilation, but there will be no added support with these breaths if SIMV is used alone. As the WOB during mechanical ventilation may be related to the resistance of the ETT , the circuit, and the ventilator, pressure or volume support may be added to augment the spontaneous breaths. Therefore, SIMV with pressure support (PS) is a frequently used mode of ventilation.

PRVC is a more recently introduced mode of ventilation, which can be provided by the newest generation of mechanical ventilators. It combines the features of both volume- and pressure-limited ventilation. Like AC modes, this mode will deliver the entire tidal breath every time the patient initiates a breath. This mode utilizes a decelerating inspiratory flow waveform (like pressure-limited ventilation on the Servo 300 and 900C ventilators) to deliver a preset VT over the inspiratory time in a pressure-limited manner. During the delivery of the tidal breath, respiratory system compliance and resistance are monitored and an algorithm is devised to deliver the VT while limiting the PIP. The ventilator regulates the inspiratory pressure up or down by as much as 3 cm H_2_ O from the previous breath to deliver the selected VT. Thus, the ventilator is continuously adapting the inspiratory pressure to changes in the resistance and compliance of the patient's respiratory system. The theory behind this mode of ventilation is that it delivers a fixed VT (as opposed to the variable VT that occurs with pressure-limited modes of ventilation) (see below). Since it works within the confines of a preset pressure limit, PRVC may limit the incidence of barotrauma that may occur when volume-limited modes of ventilation are used. To date, there are limited data demonstrating the superiority of AC, SIMV, or PRVC modes of ventilation.

### Limit variable: What controls the tidal breath (volume or pressure)?

The limit variable (pressure or volume) is that parameter which is set to determine the magnitude of the tidal breath. The type of ventilation we have chosen is named according to the limit variable: pressure-limited (pressure-control by some) or volume-limited (volume-control). In the early days of IMV, volume was generally the limit variable. A fixed VT was delivered over a fixed interval (inspiratory time) regardless of the PIP, provided that the high pressure limit was not exceeded. This in turn could lead to barotrauma.

With volume-limited ventilation, a specific VT is set by the clinician and an inspiratory time is chosen. The flow provided is then integrated based on the tidal volume and inspiratory time. For example, if a VT of 500 mL with an inspiratory time of 1 second is chosen, 500 mL will be delivered over 1 second using a gas flow of 30 L/minute (500 mL/second = 30 L/minute). In general, flow is constant during the inspiratory cycle (square-wave flow pattern). When mechanical ventilation was first applied, a VT of 10–15 mL/kg (a value 2–3 times higher than a normal tidal breath during spontaneous ventilation) was frequently used. However, more recent evidence has demonstrated that such a large VT may result in repetitive overdistention of alveoli with endothelial, epithelial, and basement membrane damage with increased microvascular permeability otherwise referred to as volutrauma.[10,11] This volutrauma may be as harmful, if not more, to the lungs than barotrauma (high pressure without the change in volume). Therefore, current practice may rely on lower tidal volumes (6–8 mL/kg) especially in patients with intrinsic lung disease and alterations in resistance and compliance of the respiratory system.

Volume-limited ventilation (in either the AC or SIMV modes) is best used in patients with normal resistance and compliance. For example, volume-limited ventilation is frequently used intraoperatively when patients with relatively normal pulmonary function receive endotracheal intubation and mechanical ventilation during surgical procedures. With poor compliance or high resistance, the higher PIP may lead to barotrauma and increased mortality in patients with ARDS and other types of pulmonary parenchymal disease.[12] The advantage of volume-limited ventilation is that a constant VT is delivered even with a changing resistance and compliance.

When volume-limited ventilation is used, the PIP should be monitored as changes in the PIP reflect changes in resistance and compliance of the respiratory system. High pressures (≥30 cm H_2_O) require an investigation which should start at the ventilator and work toward the patient including a check for kinking of the circuit or ETT, obstruction to the ETT or major airways by mucus (passing a suction catheter can frequently be used as both a diagnostic and therapeutic maneuver), auscultation to assess if there are bilateral breath sounds (to rule out mainstem intubation) and to rule out bronchospasm, a radiograph to evaluate increasing alveolar space disease (pneumonia or ARDS), or external factors impeding respiratory excursion (pneumothorax, restrictive diseases of the thorax, abdominal distention). With volume-limited ventilation, manipulation of the inspiratory time can be used to decrease the PIP as increasing the inspiratory time decreases the inspiratory gas flow rate, thereby decreasing the PIP. However, longer inspiratory times (approaching I:E ratios of 1:1) may be relatively uncomfortable for the patient who is awake since the normal I:E ratio is 1:3 or 1:4. Additionally, reversing the I:E ratio may result in air trapping and auto-PEEP. If the peak airway pressure is unacceptably high, the pressure-limited mode may be chosen (see below for a full discussion of setting the inspiratory time).

With pressure-limited ventilation, a preset pressure above PEEP is delivered over a selected inspiratory time. The inspiratory flow rate depends on the airway pressure and respiratory system compliance, achieving high levels initially and decelerating toward zero near the end of inspiration (decelerating waveform). Because inspiratory pressure is the limiting variable, changes in respiratory system mechanics (i.e., compliance and/or resistance) will result in changes in the delivered VT and minute ventilation. However, given that the PIP is controlled, the risk of barotraumas is lesser than with volume-limited ventilation. With specific types of the commonly used ICU ventilators (e.g., Servo 300), PCV is pressure-limited and time-cycled so that when the preset level of pressure is achieved, it is held for a preset time (inspiratory time), after which exhalation begins. With the newer generation of ventilators, regardless of whether pressure or volume ventilation is chosen, the type of flow waveform (square-wave or decelerating) can be chosen. Pressure-limited ventilation may be particularly beneficial in patients with decreased compliance related to high airway resistance or alveolar space disease such as pneumonia or ARDS.[12]

During pressure-limited ventilation, the delivered VT is determined by the pressure level above PEEP (sometimes referred to as the delta or ∆P), the inspiratory time, loss of VT from a leak around an uncuffed ETT, and the patient's resistance and compliance. The delta PEEP is adjusted to deliver the desired exhaled VT (generally 6–8 mL/kg). Depending on the type and manufacturer of the ventilator, the ∆P is set by either setting a PIP (VIP Bird ventilator) or pressure above PEEP (Servo 900C or 300 ventilator). In the former circumstance, it should be noted that adjusting the PEEP will affect the pressure above PEEP (the ∆P), and therefore, the VT. For example, if the PEEP is set at 5 cm H_2_ O and the PIP at 20 cm H_2_ O, the ∆P is 15 cm H_2_O. If the PEEP is increased to 7 cm H_2_O, the PIP will remain at 20 cm H_2_O, and therefore, the ∆P decreases to 13 cm H_2_O. If the PEEP and ∆P are set, increasing the PEEP by 2 cm H_2_O will also increase the PIP by 2 cm H_2_O since the ∆P remains at 15 cm H_2_O.

Apart from patients with decreased respiratory system compliance or high resistance, pressure-limited ventilation is also frequently used in neonates and infants. The delivery of a small ViT may be somewhat inaccurate based on the working parameters of the ventilator. A discrepancy of 10–20 mL in the delivered VT is not an issue when the set VT is 500 mL, but can be a significant issue when the set VT is 30–40 mL. An additional advantage of pressure-limited ventilation is the use of a decelerating flow pattern to deliver the tidal breath. Although newer ventilators allow the independent adjustment of the inspiratory flow pattern, the Servo 300 ventilator, a commonly used ventilator in the ICU population, uses a constant flow for volume-limited ventilation (square-wave pattern) and a decelerating flow for pressure-limited ventilation. The decelerating flow pattern may help in the recruitment of alveoli with long time constants (high resistance and low compliance), and thereby, over time improves the compliance. As such, it may be difficult to determine if the improved outcome of patients with ARDS who receive pressure- versus volume-limited ventilation is the result of the type of ventilation or the differences in the inspiratory flow patterns.

As with volume-limited ventilation, an inspiratory time is set with pressure-limited ventilation. As most pressure ventilators actually time-cycle (end inspiration based on the inspiratory time) and do not pressure-cycle (end inspiratory when the preset pressure is achieved), increasing the inspiratory time will increase the mean airway pressure, and hence, the exhaled VT. This is in contrast to what occurs with volume-limited ventilation where extending the inspiratory time decreases the PIP, but does not affect VT.

During pressure-limited ventilation, the exhaled VT should be monitored to assess the ongoing changes in the resistance and compliance of the respiratory system as opposed to monitoring the PIP during volume-limited ventilation. A decrease in the exhaled VT should prompt a thorough investigation into its cause that includes the same steps as outlined above for investigating an increase in PIP during volume-limited ventilation. In patients with severe lung disease, the goal of pressure-limited ventilation is to achieve a plateau pressure of less than 35 cm H_2_O. Using the plateau pressure eliminates the resistance imposed by the ETT and the major conducting airways, thereby approximating the pressures that occur within the alveoli. The plateau pressure is measured by holding a breath at the end of inspiration (there is a button on most ventilators that allow this maneuver). With a pause at the end of inspiration, the pressure within the circuit declines from the high level that occurs at the start of the breath to a baseline or plateau level.

So far, we have discussed five basic types of ventilation including AC-pressure limited, AC-volume limited, SIMV-pressure limited, SIMV-volume limited, and PRVC ventilation. These are the five basic modes of mechanical ventilation used most commonly in the ICU today. Although most modern day ICU ventilators can provide all of these modes and options, older ventilators such as the Servo 900C cannot provide SIMV-pressure limited or PRVC ventilations. With the 900C, if pressure-limited ventilation is used, it can only be performed in the AC mode.

### Inspiratory time and the inspiratory pause

The inspiratory time may be the most overlooked and underappreciated ventilator setting. Depending on the type of ventilation (pressure-limited or volume-limited), the effect of changing the inspiratory time has dramatically different effects. With pressure-limited ventilation, the delivery of the tidal breath is in actuality pressure-limited and time-cycled (the preset pressure or ∆P is held until the inspiratory time is completed). Extending the inspiration time will increase the VT. More importantly, the inspiratory time along with PEEP and PIP determines the mean airway pressure. Extending the inspiratory time increases the mean airway pressure and will thereby increase oxygenation. With volume-limited ventilation, extending the inspiratory time serves to decrease the inspiratory flow rate and thereby reduces the PIP. It can also be used as a therapeutic maneuver to help recruit alveoli with long time constants and to help the resolution of atelectasis.

With normal spontaneous ventilation, the I:E ratio is 1:3 or 1:4. The use of longer inspiratory times may be uncomfortable during spontaneous ventilation in the patient who is awake. With AC, SIMV, or PRVC ventilation, the ventilator determines the inspiratory time as opposed to supported breaths (pressure or volume support) where the patient sets the inspiratory time. In our practice, we frequently use inspiratory times of 0.3–0.5 second in infants and up to 0.7–1 second in adolescents. The use of inspiratory times that are somewhat longer than those that occur during normal tidal breathing may be helpful during acute illnesses when patients are prone to develop atelectasis while on mechanical ventilation due to lack of sighing (see below). The inspiratory time should also be adjusted based on the underlying disease process. Patients with bronchospasm and air trapping generally benefit from a shorter inspiratory time to allow for as much exhalation time as possible. Patients with alveolar space disease and poor compliance generally do better with longer inspiratory times to increase mean airway pressure and improve oxygenation. The inspiratory time can be increased up to 1.2–1.5 seconds as needed to increase the mean airway pressure and recruit alveoli, but our practice generally restricts the inspiratory time to limit the I:E at 1:1. Reversal of the I:E ratio has been used in the management of patients with severe ARDS in an attempt to augment oxygenation and allow weaning of the FiO_2_.[13] Longer inspiratory times recruit alveoli with long time constants (high resistance and low compliance), encourage collateral ventilation via pores of Cohn and canals of Lambert, reverse atelectasis, and improve the matching of ventilation and perfusion. However, longer inspiratory times especially when combined with higher ventilator rates can result in reversal of the I:E ratio which may result in inadequate exhalation times. This may result in air trapping, the stacking of one breath on another (inspiration for the next breath starts before exhalation begins) thereby resulting in auto-PEEP. An evaluation for the presence of auto-PEEP can be performed by holding the ventilator breath at the end of exhalation (there is a button on the ventilator that allows this maneuver which is otherwise known as an expiratory pause). If the airway pressure does not come down to the ventilator's level of PEEP, auto-PEEP is occurring.

Subtleties in adjusting the inspiratory time vary from one ventilator to the other. The inspiratory time may be set as a fixed time (seconds) by adjusting the inspiratory flow rate as an I:E ratio or as a percentage of the respiratory cycle. “Even when the same ventilator is used, there may even be differences in how the inspiratory time is set dependent on whether pressure or volume ventilation is used.” The inspiratory time of the VIP Bird ventilator is set by adjusting the flow rate during volume-limited ventilation and is set in seconds during pressure-limited ventilation. If the inspiratory time is set as an I:E ratio or as a percentage of the respiratory cycle, adjusting the rate will affect the actual inspiratory. For example, if the RR is set at 15 breaths/minute with an inspiratory time of 25%, it results in an inspiratory time of 1 second. Changing the rate to 20 breaths/minute with the same inspiratory time of 25% results in an inspiratory time of 0.75 seconds. Such changes can result in changes in the peak airway pressure during volume-limited ventilation or the VT during pressure-limited ventilation.

Some ventilators allow the addition of an inspiratory pause. This time is added to the end of inspiration, so its contribution to the total inspiratory time must be realized in avoiding reversal of the I:E ratio. The inspiratory pause holds the inspiratory effort at the end of inspiration without the ongoing gas flow. This maneuver serves many of the same purposes as extending the inspiratory time during pressure-limited ventilation including the recruitment of alveoli with long time constants (high resistance and low compliance), promotion of collateral ventilation via pores of Cohn and canals of Lambert, reversal of atelectasis, and improved matching of ventilation and perfusion.

### Positive end expiratory pressure

PEEP refers to positive end expiratory pressure applied during the provision of mechanical ventilation with an ETT. PEEP maintains the patency of injured lung units which may collapse during exhalation. Although physiologically accomplishing the same thing, it should be differentiated from continuous positive airway pressure (CPAP), which is applied during spontaneous ventilation. In normal adults, FRC (the volume at which lung recoil inward is balanced by chest wall recoil outward) and expiratory lung volume (ELV, the volume at which inspiration begins) are equal and exceed the CC. Thus, healthy adolescents and adults require little or no PEEP to prevent atelectasis and its associated hypoxemia from occurring. In contrast, newborns with their highly compliant chest wall will have an FRC that approaches and in some cases may be less than CC under passive (i.e., sedated, anesthetized, and/or paralyzed) conditions, thereby leading to the concept of physiologic PEEP (typically 3–5 cm H_2_O) to avoid ventilation–perfusion inequalities. This mechanical inefficiency is avoided under dynamic (i.e., spontaneously breathing) conditions because ELV is greater than FRC secondary to a rapid RR with short expiratory times (i.e., there is insufficient time for expiratory flows to reach zero; therefore, intrinsic or auto-PEEP is present), laryngeal muscle contraction during exhalation impedes expiratory airflow (this does not occur with an ETT in place), and increased intercostal muscle tone that stabilizes the chest wall thereby increasing elastic recoil. Thus, sedated or intubated infants generally require delivery of physiologic levels of PEEP to overcome the loss of these dynamic compensatory mechanisms. Higher levels of PEEP may be required in patients with alveolar space disease, increased abdominal distention, and other pathologic conditions that increase CC and decrease FRC. PEEP increases the lung volume, restores FRC, and when applied in the proper amount, improves lung compliance so that a given change in pressure results in a greater VT.

Several different methods of determining the optimal PEEP have been suggested including obtaining a chest radiograph with an evaluation of the expansion of the lung fields, increasing the PEEP to allow for an FiO_2_ of less than 0.6, performance of pressure–volume curves with increasing tidal breaths, or measurement of shunt fraction using a flow-directed pulmonary artery catheter. In the pressure–volume curves (volume on the y axis and plateau pressure on the x axis), the curve will be sigmoidal in shape with a marked increase in the volume achieved with small changes in pressure initially noted at a lower plateau pressure (lower inflection point) and then a decrease in these volumes at a higher plateau pressure (upper inflection point).[14,15] Some clinicians use the lower inflection point to determine the level at which PEEP is set.

In most instances, a change in PEEP is the first step used for regulating mean airway pressure in patients with lung disease thereby moving to the steep portion of the pressure–volume curve and restoring normal compliance of the respiratory system. The application of PEEP prevents airway pressure from dropping below critical closing pressure (maintaining airway patency and alveolar volume throughout the ventilatory cycle), redistributes pulmonary edema fluid from alveoli to the interstitium, maintains alveolar surfactant activity, and improves ventilation to low V/Q lung units.[16,17] Excessive levels of PEEP can be counterproductive in patients with ARDS as they increase deadspace ventilation, depress cardiovascular function, decrease alveolar compliance via excessive distention, and increase pulmonary vascular resistance.

### RR and fraction of inspired oxygen concentration

The final two decisions regarding ventilatory parameters are somewhat self-explanatory. Immediately before and for a brief period following endotracheal intubation, most patients are ventilated with an FiO_2_ of 1.0. Depending on the severity of the underlying lung injury, this can generally be rapidly weaned according to the oxygen saturation on the pulse oximeter without the need for arterial blood gas analysis. The risk of toxic effects of oxygen is minimized by using the lowest FiO_2_ which results in an oxygen saturation of 90% or a PaO_2_ of 60 mmHg. In patients who cannot be weaned to an FiO_2_ less than 0.5–0.6, other maneuvers to increase oxygenation (increasing the mean airway pressure by increasing PEEP or inspiratory time) should be attempted. Additionally, accepting lower oxygen saturations (80–90%) may be acceptable in patients with severe lung injury. With the use of sedatives, judicious transfusion to increase hemoglobin levels, and control of peripheral oxygen consumption, adequate oxygen delivery to meet tissue can usually be maintained with an oxygen saturation of 80%.

The ventilator rate is set primarily based on the patient's age, the desired PaCO_2_, and the VT that is delivered. In patients with severe lung injury, higher rates are used to compensate for the lower VT thereby limiting ventilator-induced lung injury. Guidelines for starting ranges of RRs include 10–12 breaths/minute for adults or adolescents, 12–16 breaths/minute for older children (6–10 years of age), 16–24 breaths/minute for toddlers, and 24–30 breaths/minute for neonates and infants. Higher rates may be needed in patients with more severe degrees of lung injury, when hyperventilation is used to treat increased intracranial pressure or pulmonary hypertension, or if endogenous CO_2_ production is elevated. However, ventilation to normocapnia is not always required, as modern therapy for ARDS and other types of acute lung injuries employs permissive hypercapnia where hypercarbia is allowed provided the pH is greater than 7.25.

### Supported ventilation

The pressure–volume (compliance) and pressure-flow (resistance) characteristics of the respiratory system determine the WOB. Physiologic factors including the impendence from the elastic recoil of the lung and chest wall and the frictional resistance to gas flow in the airways combined with mechanical factors (presence of an ETT or valves in the ventilator) can both further contribute to the WOB. Various disease processes through either a decrease in respiratory compliance (alveolar space disease from pneumonia or ARDS) and/or an increase in respiratory resistance (bronchospasm or upper airway lesions) can also increase the WOB.

Supported ventilation is defined as a breath that is triggered by the patient, limited by the ventilator (volume or pressure), and cycled by the patient (the patient determines the inspiratory time). It is used with SIMV to support spontaneous breaths that occur between ventilator breaths and thereby limits WOB. It can also be used with controlled ventilation as a means of weaning patients from mechanical ventilation. Ventilation is spontaneous in nature because the patient determines the ventilatory pattern (i.e., frequency, inspiratory, and expiratory times) by initiating and terminating each breath. Therefore, supported ventilation is only used in patients with an intact ventilatory drive. With this form of ventilation, the patient provides the work to trigger the breath and then interacts with the ventilator to perform a variable amount of the remaining work with each breath.

Pressure support ventilation (PSV) is a mode in which the patient triggers the ventilator to deliver a flow of gas sufficient to provide a preset pressure level. The breath is terminated when inspiratory flow decreases to a percentage (generally 25%) of its peak value rather than by volume, pressure, or time. At that point, the exhalation valve closes to pressurize the circuit to the predetermined expiratory limit (PEEP). Therefore, the patient retains control of the cycle length and flow characteristics. The VT is determined by the patient's inspiratory effort, the preset pressure limit, and respiratory system impedance (resistance and compliance). PSV is used most commonly to compensate for the inspiratory work imposed by the ETT.[18,19] PSV can prevent diaphragmatic fatigue in patients who failed to wean from conventional ventilation, possibly due to changes in the pressure–volume characteristics and enhanced endurance training of the diaphragm.[20,22]

Two different methods of weaning ventilation with PSV have been advocated. One approach involves setting the pressure limit high enough to achieve delivery of the typical mechanical tidal breaths (6–8 mL/kg) with no back-up SIMV rate and then gradually decreasing the pressure down to the minimum value needed to overcome the imposed work of the ETT and ventilator circuit prior to extubation. The second method involves the combined use of SIMV and PSV in which the pressure limit during PSV breaths is set to minimize the imposed work of the ETT and circuit only. The SIMV rate is then gradually decreased to 0–4, at which time the ETT is removed. Controlled studies have suggested that PSV weaning is more effective than SIMV weaning in adult patients who are difficult to liberate from mechanical ventilation.[23,24] Similar data in the pediatric population are not available.

Volume support ventilation (VSV) is a newer mode of ventilation in which supported breaths are volume-limited while using a decelerating inspiratory flow that is flow-cycled as with PSV. With this mode of ventilation, the pressure assist is regulated to deliver the preset volume, provided a maximum pressure limit is not exceeded, with each supported breath. This mode has all of the theoretical benefits of PSV (the patient controls the inspiratory flow, time, and frequency) with the unique capability of providing a guaranteed minimum minute volume. To date, the experience with this mode of supported ventilation in children is limited.[25]

### Weaning of mechanical ventilation

Appropriate weaning techniques are mandatory as complications related to mechanical ventilation and endotracheal intubation are generally dependent on the duration of support.[26] For the vast majority of patients requiring mechanical ventilatory assistance, weaning of support is not problematic. However, regardless of the clinical scenario, there will a small percentage of patients who may be difficult to wean from positive pressure ventilation or who fail tracheal extubation. The success of weaning ventilatory support is dependent upon the consideration of the patient's general status with correction of endocrine (hypothyroidism) or electrolyte imbalances (hypokalemia, hypophosphatemia) which may affect weaning, the maintenance of adequate ventilatory reserve, and the attainment of favorable respiratory mechanics. In most cases, the maintenance of adequate nutrition during the period of mechanical ventilation and acute illness is mandatory to maintain adequate strength of the respiratory musculature. Clinicians working with patients requiring mechanical ventilation have attempted for years to define objective measures, which may predict the success or failure of discontinuation of mechanical ventilatory support in patients undergoing positive pressure ventilation [Table 4]. As a full review of this topic is beyond the scope of this study, the reader is referred to[27] for a full review of this subject. The criteria for weaning from mechanical ventilation and tracheal extubation are typically directed at ensuring adequate gas exchange (oxygenation and ventilation) as well as the presence of sufficient ventilatory reserve to maintain prolonged respiratory function without mechanical support. Majority of the data are from adults with respiratory failure and many of the criteria have high false positive and/or negative rates. Many of the criteria are based on an instantaneous measurement when in fact many patients fail extubation due to increasing respiratory muscle fatigue over hours or days. Integration of several physiologic variables such as respiratory frequency/VT, also known as the rapid shallow breathing index (RSBI), have recently shown promise as a more accurate predictor of success to wean from ventilation in adults.[28]

**Table 4:** Proposed criteria for weaning of mechanical ventilation

Central nervous system
Adequate ventilatory drive
Adequate airway protection and clearance of secretions
Adequate mental status and responsiveness
Airway
Adequate subglottic space (airleak at <25–30 cm H_2_O with cuff
Deflated or uncuffed ETT)
Musculoskeletal system
Negative inspiratory force better than –30 cm H_2_O
Forced vital capacity > 15–20 mL/kg
No residual neuromuscular blockade
Respiratory system
Adequate ventilatory function
Spontaneous tidal volume > 6 mL/kg
Rapid shallow breathing index < 8
Adequate oxygenation with PEEP < 5 cm H_2_O
PaO2 > 70 mmHg with FiO_2_ < 0.4
PaO_2_/ FiO_2_ < 200

With a lack of formal studies to demonstrate the superiority of one technique over another, there are several methods of weaning patients from mechanical ventilation. In the pediatric population, SIMV remains a commonly used method for weaning of mechanical ventilatory support. This mode of ventilation allows for a gradual decrease in the amount of minute ventilation delivered by the ventilator by slowly decreasing the set RR as tolerated by the patient. In general, the frequency of positive pressure breaths is decreased in 2–4 breaths/minute increments followed by an assessment of the patient's status. This is frequently done by clinical evaluation by following the patient's RR, oxygen requirement, and evaluating signs of respiratory distress. The clinical assessment is frequently supplemented by the use of noninvasive measures of oxygenation and ventilatory status (pulse oximetry, end-tidal CO_2_ and transcutaneous CO_2_ monitoring). Once the rate has been weaned to 0–4 breaths/minute, the levels of PS and PEEP are also weaned. PS is weaned to a level that is thought to provide only enough support to overcome the WOB imposed by the ETT, the circuit, and the ventilator. No formal studies exist to demonstrate the exact level of PS required to achieve this goal; however, levels of 6–10 cm H_2_ 0 are generally accepted as providing minimal extra support in the pediatric age group. Higher levels may provide additional support thereby suggesting that the patient can be removed from mechanical ventilation when in fact there is still a significant amount of support provided by the ventilator. When the patient has reached some arbitrary minimum SIMV rate (i.e., 0–2 in children, 4 in toddlers, and 4–8 in infants), they are ready for a trial of extubation.[29] More recently, a spontaneous breathing trial without PS (T-tube or CPAP trial) has been suggested prior to tracheal extubation.[27] Given the low flow rates generated during spontaneous breathing, it has been suggested that such techniques do not impose excessive WOB on the patient.

When the AC mode is used for weaning, both the rate and the level of support (volume or pressure) must be weaned. The AC mode may be beneficial in neonates and infants who have difficulty triggering the PS mode of some ventilators. The baseline amount of support that is acceptable prior to attempted extubation should be an amount estimated to provide only enough support to overcome the WOB imposed by the ETT and ventilator. As with PS ventilation, there are limited data in children to determine the exact level of support needed to compensate for the WOB added by the ETT and the ventilator.

## CONCLUSIONS

The ability to implement and provide mechanical support to patients with respiratory failure may be the single most important maneuver ever added to the ICU armamentarium. Although this technology started with the introduction of endotracheal intubation in the operating room, the more recent advances have centered on the design and function of modern day mechanical ventilators as well as an improved understanding of ventilator-induced lung injury. Prospective clinical studies have delineated important techniques that may improve survival and limit morbidity in patients with acute lung injury. Despite these advances, patients may fail conventional mechanical ventilation and require other modalities of support including high-frequency ventilation or extracorporeal support.
